# Impact of Infant Thoracic Non-cardiac Perioperative Critical Care on Homotopic-Like Corpus Callosum and Forebrain Sub-regional Volumes

**DOI:** 10.3389/fpain.2022.788903

**Published:** 2022-04-07

**Authors:** Mackenzie Shea Kagan, Chandler R. L. Mongerson, David Zurakowski, Dusica Bajic

**Affiliations:** ^1^Department of Anesthesiology, Critical Care and Pain Medicine, Boston Children's Hospital, Boston, MA, United States; ^2^Harvard Medical School, Boston, MA, United States

**Keywords:** brain, esophageal atresia, long-gap esophageal atresia, LGEA, morphometric, MRI, pediatric, premature

## Abstract

Previously, we reported quantitatively smaller total corpus callosum (CC) and total forebrain size in critically ill term-born and premature patients following complex perioperative critical care for long-gap esophageal atresia (LGEA) that included Foker process repair. We extended our cross-sectional pilot study to determine sub-regional volumes of CC and forebrain using structural brain MRI. Our objective was to evaluate region-specific CC as an *in-vivo* marker for decreased myelination and/or cortical neural loss of homotopic-like sub-regions of the forebrain. Term-born (*n* = 13) and premature (*n* = 13) patients, and healthy naïve controls (*n* = 21) <1-year corrected age underwent non-sedated MRI using a 3T Siemens scanner, as per IRB approval at Boston Children's Hospital following completion of clinical treatment for Foker process. We used ITK-SNAP (v.3.6) to *manually* segment six sub-regions of CC and eight sub-regions of forebrain as per previously reported methodology. Group differences were assessed using a general linear model univariate analysis with corrected age at scan as a covariate. Our analysis implicates globally smaller CC and forebrain with sub-region II (viz. rostral body of CC known to connect to pre-motor cortex) to be least affected in comparison to other CC sub-regions in LGEA patients. Our report of smaller subgenual forebrain implicates (mal)adaptation in limbic circuits development in selected group of infant patients following LGEA repair. Future studies should include diffusion tractography studies of CC in further evaluation of what appears to represent global decrease in homotopic-like CC/forebrain size following complex perioperative critical care of infants born with LGEA.

## Highlights

- Infants born with long-gap esophageal atresia (LGEA) have decreased forebrain and corpus callosum volumes following Foker process repair
- We report global decrease in homotopic-like CC/forebrain size following complex perioperative critical care of infants born with LGEA
- Decreased non-homotopic subgenual region of the forebrain implicates (mal)adaptation in limbic circuits development in studied group of infant patients.

## Introduction

The corpus callosum (CC) represents the major and largest commissural tract in the human brain connecting two cerebral hemispheres. Indeed, callosal axons originate primarily from neurons in layer II/II and layer V of the neocortex (1, 2). Those callosal axons are arranged in topographically ordered manner within the CC, before their projection into their corresponding contralateral cortical areas (3). As such, the CC is divided into a total of six anatomical sub-regions that correspond to topographically ordered sub-regions of forebrain (4), also described as homotopic relationship (5). Development of the CC and its projections in the forebrain occurs rapidly in the third trimester of pregnancy and throughout the first year of life (6, 7). Prematurity and/or critical illness is associated with hindered CC/forebrain development and disruption of subsequent developmental processes (8, 9). As CC myelination has been shown to occur anteriorly to posteriorly (10), we seek to evaluate sub-region-specific CC and forebrain volumes as to identify potential early structural vulnerability that can occur in the context of the complex perioperative critical care in infancy.

**Figure 1:**
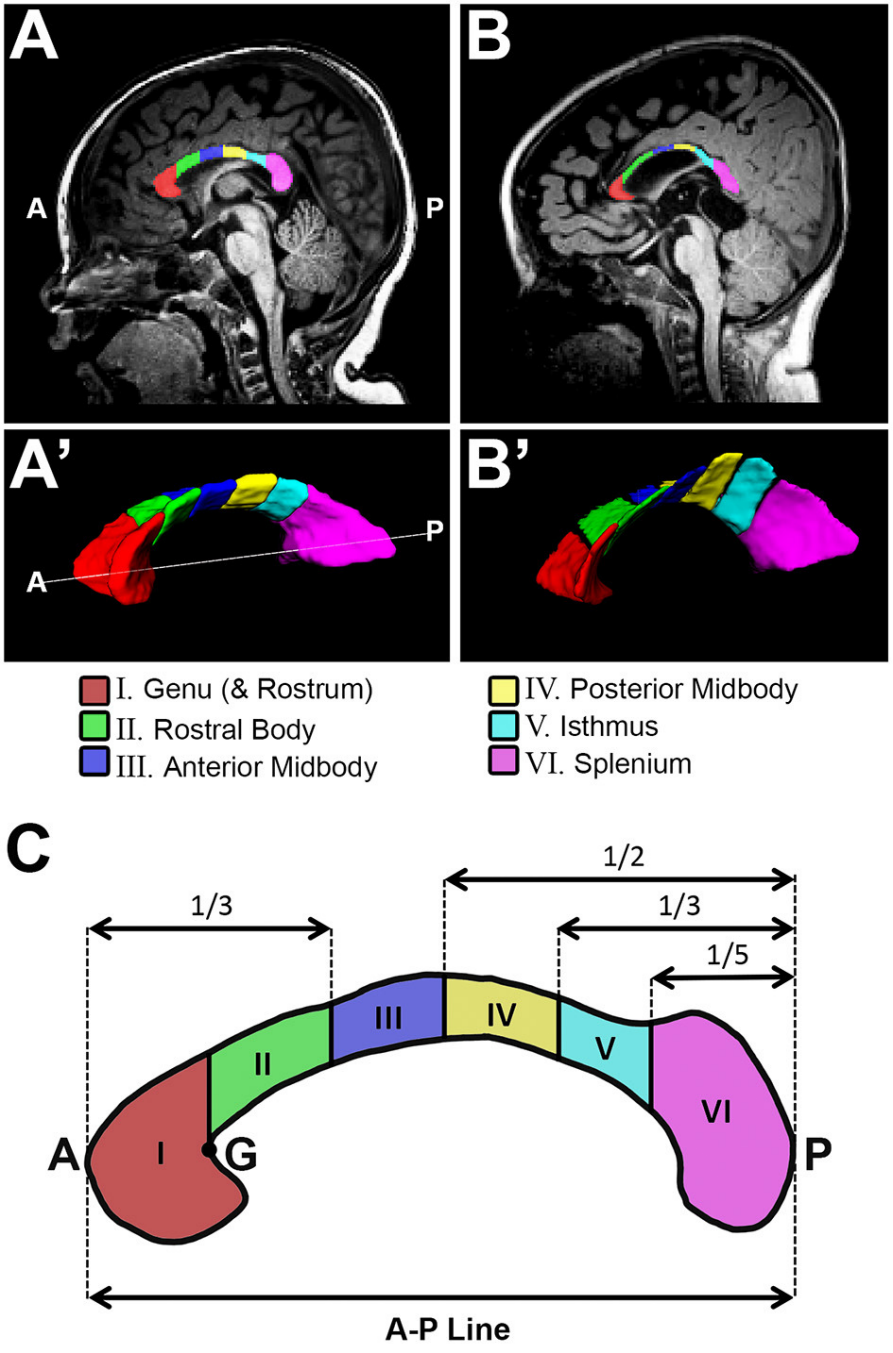
Sub-regional corpus callosum analysis. Representative T1-weighted corpus callosum (CC) segmentation for a term-born otherwise healthy control **(A,A')** and a term-born patient **(B,B')** scanned at 10 and 9 months of age, respectively. Volumes of CC were estimated for a total of 6 CC sub-regions **(C)**. Sub-regional segmentation used the proportion-based parcellation scheme originally proposed by Witelson (23), with slight modification based on Venkatasubramanian et al. (37) (see Methods for details). A, anterior; G, genu; P, posterior.

Our recent pilot study identified a group of critically ill infants born with long-gap esophageal atresia (LGEA) to have smaller corpus callosum (11) and forebrain (12) volumes following complex perioperative critical care. Unlike *short-gap* defects that can be repaired by direct anastomosis (13), infants born with long esophageal disconnects (>3 cm) underwent complex perioperative critical care involving tension-induced esophageal growth known as the Foker process (14–16) requiring prolonged sedation ≥5 days (17–19) leading to physical dependence to the drugs of sedation (17, 20, 21). Such complex perioperative critical care spans period of weeks, as described previously [Figure 1 in (19, 22)].

We hypothesized that when compared to healthy infants, both critically ill term- and preterm-born patients following perioperative critical care for LGEA repair with Foker process would exhibit (mal)adaptation in sub-region-specific CC and forebrain volumes. Thus, our novel analysis aimed to quantify homotopic-like CC and forebrain sub-regional volumes using T1-weighted brain MRI in our recent pilot cohort of infants following repair of LGEA (22). Our broader objective was to evaluate sub-region-specific CC volume as a possible *in-vivo* marker for decreased myelination and/or cortical neural loss of homotopic sub-regions of the forebrain. We used previously published methods of semi-automated segmentation of the CC and forebrain that relied on distinct anatomical landmarks in the infant brain, as described by Witelson (23) and Peterson et al. (24, 25), respectively. Anatomically divided sub-regions of the CC and forebrain were schematically related to each other according to the existing literature and in the spirit of a homotopic relationship (26). Our preliminary data were previously presented only as virtual abstract at a conference (27).

## Methods

### Study Design and Participants

Our report builds on our previous pilot infant MRI study that received ethical approval from Institutional Review Board as a “no more than minimal risk” study. Specifically, we extended our previous T1-weighted brain MRI analysis of total CC volumes (11) and T2-weighted analysis of total forebrain volumes (12) to the current T1-weighted analysis of sub-regional volumes of both CC, as well as that of the forebrain. Since presented data is from the same infant study subjects, previously described methodological approach (11, 22) for (1) recruitment criteria and (2) MRI scanning process apply to this study as well. Briefly, informed written parental consent was obtained prior to subject participation, in accordance with the Declaration of Helsinki and Good Clinical Practice guidelines. The family of each subject received a $90 gift card for participation in the non-sedated research brain MRI scan.

#### Patients With LGEA

Eligibility criteria included both term-born [37–42 weeks gestational age (GA) at birth] and preterm-born patients <1 year gestation-corrected age. The preterm-born group included only very preterm (28 to < 32 weeks GA) and moderate or late preterm-born infants (32 to < 37 weeks GA) *as defined by The World Health Organization* (28). All patients underwent initial LGEA repair at either Boston Children's Hospital or an outside institution in the first 2 months of life. Specifically, Foker process (14–16) allows for tension-induced esophageal growth for long esophageal atresia disconnects (viz. > 3 cm found in LGEA). Such complex perioperative care involves several stages (29): (1) Foker I thoracotomy to place traction sutures onto blind esophageal ends; (2) Esophageal lengthening by continuous traction on the esophagus pouches to induce esophageal tissue growth which requires sedation; (3) Foker II thoracotomy to approximate esophageal ends and perform primary esophageal anastomosis; (4) Post-Foker healing of the anastomosis with weaning of sedation and transition from total parenteral nutrition to enteral feeds prior to hospital discharge. The unique aspect of such complex perioperative care in infancy is not only related to repeated anesthesia and surgery, but to prolonged sedation exposure ≥5 days known to be associated with development of physical dependence to the drugs of sedation (17, 20, 21). Representative timeline illustrating sequence of perioperative critical care for LGEA repair was presented previously (17–19). Associations between individual MRI end-point measures (e.g., number of cranial MRI findings and brain volumes) and the clinical measures of care as to assess the severity of underlying disease in cohort patients will be presented elsewhere.

Exclusion criteria included: (1) extreme prematurity (<28 weeks GA); (2) diagnosis of small for gestational age and/or intrauterine growth restriction (SGA/IUGR) (30, 31); (3) extracorporeal membrane oxygenation exposure; (4) clinically indicated cranial ultrasound findings (e.g., ventricular enlargement with or without gray matter and/or ventricular hemorrhage); (5) neurological disease as documented in clinical record (e.g., seizures); (6) chromosomal abnormalities (e.g., Down's syndrome); (7) prenatal drug exposure to either drugs of abuse or prescription medications; and/or (8) MRI incompatible implants. Indeed, we recruited only those patients born with LGEA that had no clinical evidence of neurological problems at the time of recruitment as per detailed chart review and/or cranial ultrasound findings when available.

#### Term-Born Controls

Healthy term-born infants (<1 year old) with no prior exposure to surgery, anesthesia, or sedation (i.e., naïve to perioperative treatment) were recruited from a pool of Boston Children's Hospital outpatients and two neighboring newborn centers (Beth Israel Deaconess Medical Center and Brigham and Women's Hospital, Boston, MA). These controls served as a reference baseline for typical sub-regional CC and forebrain size. This study differs from our previous T1-weighted analysis [Table 1 in (11)] by addition of one extra control infant (total *n* = 21). An updated summary of final group characteristics is shown in Table 1.

**Table 1:** Pilot study recruitment and characteristics.

	**Term-born controls** **(*n* = 21)**	**Term-born patients** **(*n* = 13)**	**Preterm-born patients** **(*n* = 13)**
**Recruitment process**			
Considered/(chart) reviewed	63	173	108
Eligible (%reviewed)	60 (95%)	63 (36%)	49 (45%)
Approached (%eligible)	57 (95%)	40 (63%)	23 (47%)
Consented (%approached)	23 (40%)	19 (48%)	18 (78%)
Scanned (%consented)	23 (100%)	13 (68%)	13 (72%)
Included/analyzed (%scanned)	21 (91%)	13 (100%)	13 (100%)
**Group characteristics**			
Sex (male), *n* (%)	17 (81%)	7 (54%)	8 (62%)
GA at birth (weeks), Mean ± SD	39.3 ± 1.1	38.5 ± 1.1	32.2 ± 2.9
CA at scan (months), Median (range)	4.5 (0.5–12.3)	5.4 (0.7–13.0)	3.8 (1.4–7.5)
Twin births, *n* (%)	1 (5%)	1 (8%)	2 (15%)
**Primary diagnoses**			
Isolated LGEA, *n* (%)	0	3 (23%)	3 (23%)
LGEA with TEF, *n* (%)	0	5 (38%)	9 (69%)
Other, *n* (%)	0	5 (38%)	1 (8%)

*Table summarizes demographic and clinical characteristics for the 3 groups (term-born controls, and term- and preterm-born patients) included in T1-weighted MRI analysis. The preterm-born group included only very preterm (28 to <32 weeks GA) and moderate or late preterm infants (32 to <37 weeks GA) as defined by The World Health Organization (28). Group numbers are updated since our previous publication [Table 1 in (11)] to include one additional control subject. In addition to isolated long-gap esophageal atresia (LGEA) and LGEA with tracheo-esophageal fistula (TEF), a few patients had other non-cardiac congenital anomaly diagnoses that included LGEA as part of VACTERL association (without cardiac component). Typically, infants diagnosed with VACTERL exhibit ≥3 of the characteristic features (viz. Vertebral defects, Anal atresia, Cardiac defects, Tracheo-Esophageal fistula, Renal anomalies, and Limb abnormalities). None of the infants included in analysis had cardiac anomalies requiring surgery, nor exposure to extracorporeal membrane oxygenation. For other exclusion criteria, see Methods. GA, gestational age; CA, corrected age*.

### MRI Acquisition

All infant patients underwent non-sedated research brain MRI at a point in time just before the hospital discharge following completion of initial surgical repair, or during subsequent admissions for follow up management in the 1st year of life. Thus, subjects were scanned throughout the infancy, depending on the time of their recruitment. A “feed and wrap” approach was used on all infants undergoing a non-sedated research brain MRI scan after the completion of complex perioperative treatment (32–35). Corrected age at scan for all cohort subjects was calculated as follows: postnatal age (weeks) – [40 – GA at birth (weeks)]. Please, refer to our previous publication in regard to individual total CC [Figure 5 in (11)] and total forebrain volumes [Figure 3 in (12)] with respect to corrected GA at scan. Patients were scanned at late evenings or nights using a 3T TrioTim MRI system equipped with 32-channel receive-only head coil and body-transmission (Siemens Healthcare Inc., USA) as per previously described protocol (12, 22). Foam earplugs (Newmatic Medical, Birmingham, AL) and earmuffs (MRI-Safe Neonatal Noise Guards, Universal Medical, Norwood, MA) were used for noise protection. Smaller infants were further supported using beanbags (viz. gentle vacuum bag immobilizer), while infants older than 3 months were allowed to assume a more relaxed position (e.g., arms next to face). Despite undergoing a non-sedated research scan, due to complexity and severity of clinical status, a single physician (DB) continuously monitored all infants for a stable heart rate and oxygenation throughout the MRI acquisition. Structural T1-weighted images were acquired using a MPRAGE sequence (repetition time = 2.52 s; echo time = 1.74 ms; flip angle = 7°; field of view = 192 × 192 mm^2^; voxel size = 1 × 1 × 1 mm^3^; 144 sagittal slices). We collected 100% of T1-weighted images for all patients (*n* = 13/group), and 91% (*n* = 21/23) for term-born controls (Table 1). Regarding the latter, one infant had partial brain coverage that precluded analysis of total brain volume (*n* = 20 controls for forebrain analysis) but allowed for CC analysis (*n* = 21 controls). Although we noted ringing artifact due to motion only in 1/21 controls and 1/13 premature patients, it was mild enough not to require motion correction or obscure tissue segmentation.

### Infant Brain Segmentation of T1-Weighted MRI Data

**Figure 2:**
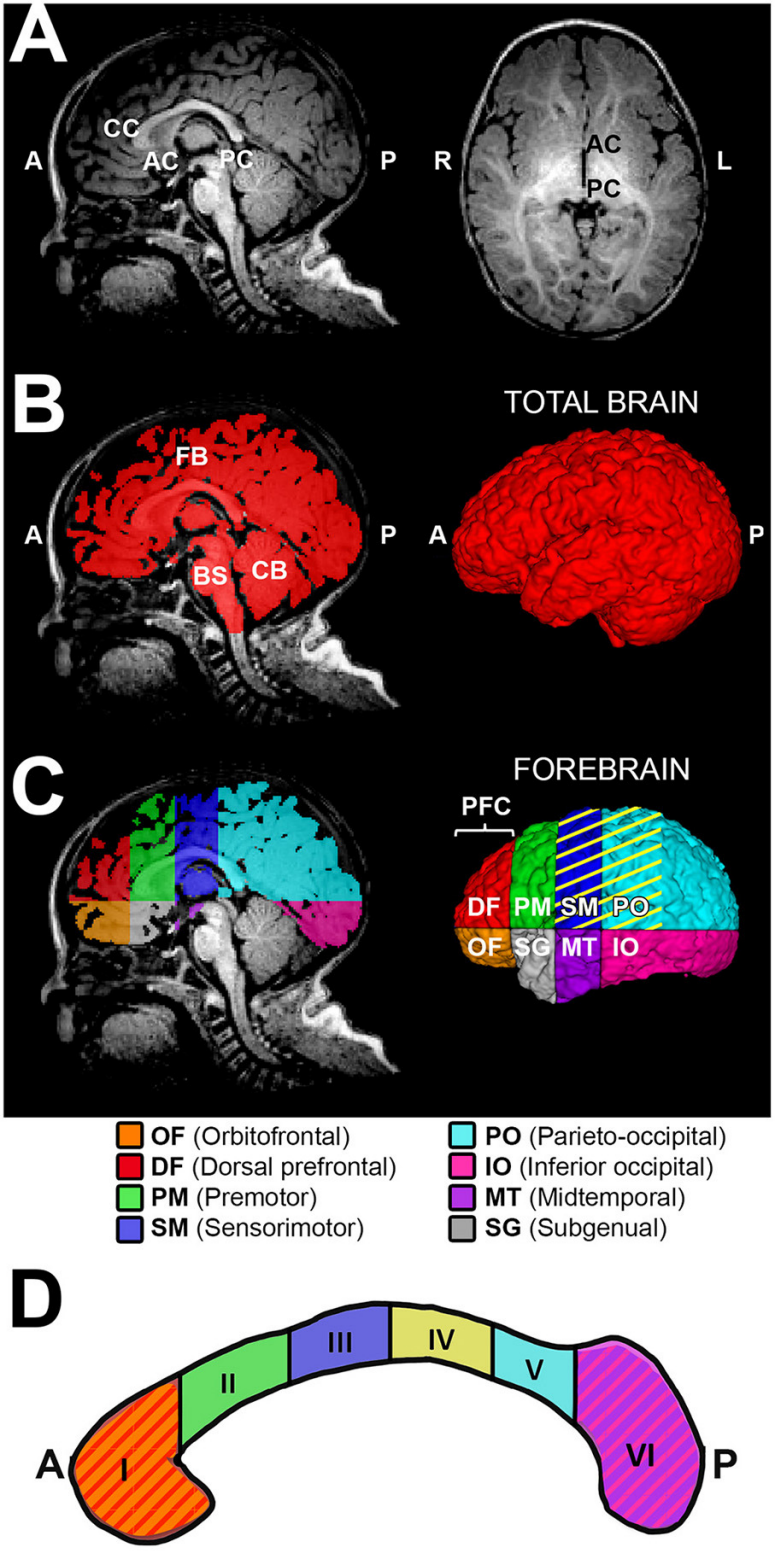
Sub-regional forebrain analysis. T1-weighted regional forebrain analysis is shown for a term-born otherwise healthy control scanned at 7 months of age. **(A)** shows T1 image in midsagittal (left) and axial (right) views, noting the key anatomical landmarks used for sub-regional forebrain analysis [the anterior-most aspect of corpus callosum (CC), the anterior commissure (AC), and the posterior commissure (PC)]. Following total brain segmentation **(B)**, the cerebellum (CB) and brainstem (BS) were manually erased to create a mask of total forebrain. Subsequently, forebrain was parcellated into eight sub-regions **(C)**, as per Peterson et al. (24). Orbitofrontal (OF; orange) and dorsal prefrontal (DF; red) sub-regions were combined and analyzed as the prefrontal cortex (PFC) sub-region. Color pattern in the corpus callosum outline **(D)** schematically illustrates the homotopic-like relationship between different sub-regions of CC and forebrain according to existing literature (26). Specifically, CC region I (genu and rostrum) projects to PFC, region II (rostral body) projects to PM, region III (anterior midbody) projects to SM, region IV (posterior midbody) projects to SM and PO (**C**, yellow stripes), region V (isthmus) projects to PO, and region VI (splenium) projects to IO and MT. The subgenual (SG) area of forebrain is predominantly comprised of limbic structures, with no major direct projections from the CC. A, anterior; L, left; P, posterior; R, right.

Prior to any tissue segmentation, Freeview (v.2.0) from Freesurfer (the General Hospital Corporation, Boston, MA) was used to correct for any head tilt during MRI acquisition. In other words, preprocessing of T1-weighted images included alignment along the anterior commissure - posterior commissure (AC-PC) line [see Figure 2 in (22)]. A single rater with neuroanatomical expertise blindly performed tissue segmentation, which was subsequently checked by a senior researcher for internal consistency. Specifically, ITK-SNAP software (v.3.6.0; www.itksnap.org) (36) was used for total and sub-regional CC segmentation (Figure 1) and total brain and forebrain segmentation (Figure 2A–C) of T1-weighted images. We also performed surface area analysis, but since finals 2-D data results did not significantly differ from volumetric data, 2D results are not presented.

#### CC Segmentation

**Figure 3:**
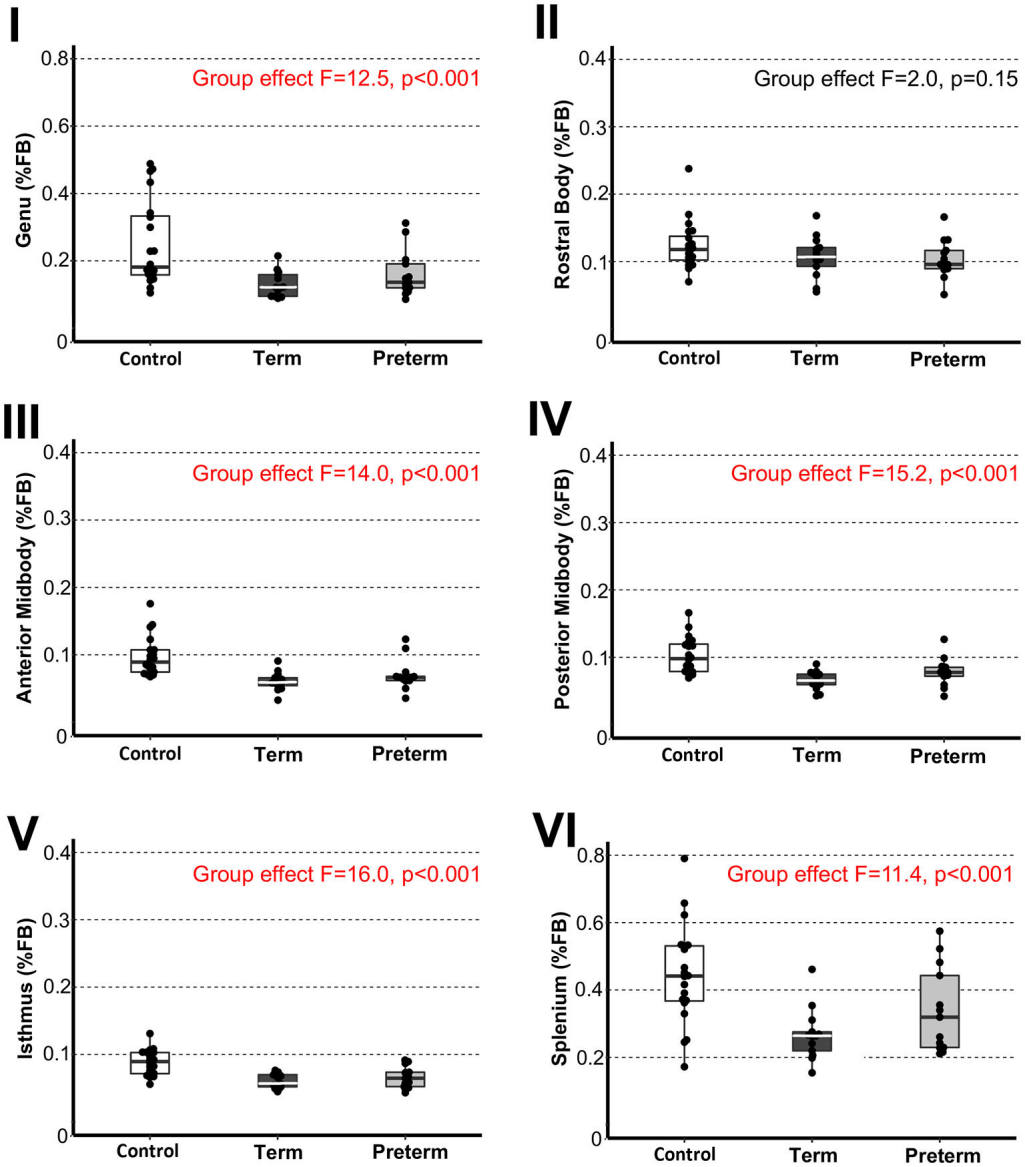
Sub-regional corpus callosum volumes. Graphs display individual *normalized* sub-regional volumes as a % total forebrain (FB) volume for the three study groups: (1) term-born controls (*n* = 20; white box), (2) term-born patients (*n* = 13; black box), and (3) preterm-born patients (*n* = 13; gray box). As noted in Methods section, forebrain volume used for normalization was not available for 1/21 controls due to incomplete brain coverage, which accounts for final control group *n* = 20. Normalized volumes as % FB of all CC sub-regions, except for the rostral body, were significantly smaller in both term-born and preterm patients compared to controls (*p* ≤ 0.002; Table 2), but not between patient groups. Dots represent individual data values, boxes represent the interquartile range (25–75%), and solid horizontal lines represent medians. Roman numerals for each graph also depict classification of CC sub-regions as outlined in the Figure 1.

We previously described a detailed method for manual tracing of T1-weighted data for total CC volume (cm^3^) segmentations [Figure 3 in (11)]. The CC sub-regional parcellation scheme used in this study is based on previously established functional topographical division across the white matter tract (38). Furthermore, manual parcellation of the CC sub-regions was done in accordance with previously validated approach by Witelson (23), with slight modifications based on Venkatasubramanian et al. (37). Specifically, we performed sub-regional CC segmentation (Figure 1) as follows:

##### CC Volumetric Segmentation

Manual CC segmentation (Figure 1A',B') was done in line with previously described protocol for CC segmentation in newborns by Yu et al. (39) to exclude encircling cerebral vasculature and neighboring tissues including the subcallosal area, cingulum, and fornix (columns, body, and crux). Previously, we extensively described and illustrated [Figure 3B–F in (11)] lateral boundaries of CC segmentation: lateral outermost edges of the CC were bounded by the anterior and posterior corona radiata (40).

##### Sub-regional CC Segmentation

Illustration of parcellation scheme for sub-regional CC division is shown in Figure 1C. As previously described [Figure 2A in (11)], a linear segment running from the most anterior tip of the genu and the most posterior point of the splenium in the mid-sagittal section (Figure 1A,B) was used to create an anterior – posterior (A-P) line that represents CC length in the sagittal view (Figure 1C). ITK-SNAP software (v.3.6.0; www.itksnap.org) (36) was used to measure this A-P length (cm) of the CC. A-P line served for subsequent sub-regional segmentation of CC (Figure 1C). Specifically, regional boundaries were established along five coronal planes corresponding to the crook of the genu (Figure 1C - point “G”), as well as at intervals of one third, one half, two thirds, and four fifths the total length of the A-P line. This resulted in parcellation of 6 CC sub-regions as follows: (I) genu (includes rostrum), (II) rostral body (III) anterior midbody, (IV) posterior midbody, (V) isthmus, and (VI) splenium. In alignment with previous literature (41), rostrum was included as part of the genu segmentation given its very small size and highly variable shape in infancy.

#### Forebrain Segmentation

We adapted aspects of our method of total brain and forebrain segmentation previously used for T2-weighted analysis (12, 22) to T1-weighted MRI data used in this study. Briefly, we performed the semi-automated approach as follows:

##### Total Brain and Forebrain Volumetric Segmentation

Total brain volume required the following semi-automated tissue segmentation steps: (i) Skull-stripping of T1-weighted images via manual tracing of the whole brain outline (Figure 2B); and (ii) Partial volume segmentation of cerebrospinal fluid (CSF) using **F**MRIB's **A**utomated **S**egmentation **T**ool (FAST) (42). We used tools in **F**MRIB **S**oftware **L**ibrary (FSL; v.5.0) to eliminate ventricular system included in the whole brain mask. Specifically, CSF partial volume estimate was (a) thresholded at 99% (eliminating voxels with <99% of their volume comprising CSF), (b) converted to a binary mask, and (c) subtracted from a mask of the whole brain outline in order to generate mask of total brain volume *excluding* the ventricular system, which underwent additional (d) minor manual editing to draw-in any missing brain tissue, resulting in a finalized total brain mask (Figure 2B). T1-weighted data analysis of total brain volumes in infant with LGEA has been previously published (43) while total brain volume masks served for total and sub-regional forebrain segmentation in this study. Total forebrain tissue mask was created by simply erasing the cerebellum and brainstem (medulla, pons and midbrain) from a total brain mask (Figure 2B,C).

##### Sub-regional Forebrain Segmentation

Due to ongoing development and myelination throughout a patient's first year of life, there is a decreased gray to white matter tissue contrast in infant brains (44) that poses challenges for automated infant brain segmentation. We selected sub-regional analysis of the forebrain based on previously published scheme by Peterson et al. (24, 25). The reliability and validity of related schemes of brain subdivision have been previously documented (24, 25, 45). Each forebrain mask was divided into eight anatomical sub-regions using one axial plane containing the AC-PC line, and three coronal planes (tangent to genu of CC, the anterior commissure, and the posterior commissure; Figure 2A,C). Thus, the eight forebrain sub-regions, as illustrated in Figure 2C, were segmented: orbitofrontal (OF), dorsal prefrontal (DF), premotor (PM), sensorimotor (SM), parieto-occipital (PO), inferior occipital (IO), midtemporal (MT), and subgenual (SG). Given the small size of the two most anterior sub-regions – the orbitofrontal and the dorsal prefrontal sub-regions were combined and defined as the prefrontal cortex (PFC) sub-region. This resulted in a total of seven forebrain sub-regions included in the final analysis. Note that majority of terminology of each forebrain sub-region is related to the cortical nomenclature, although listed sub-regions encompass the cortex and subcortical tissue of the forebrain.

### Structural Quantification

**Figure 4:**
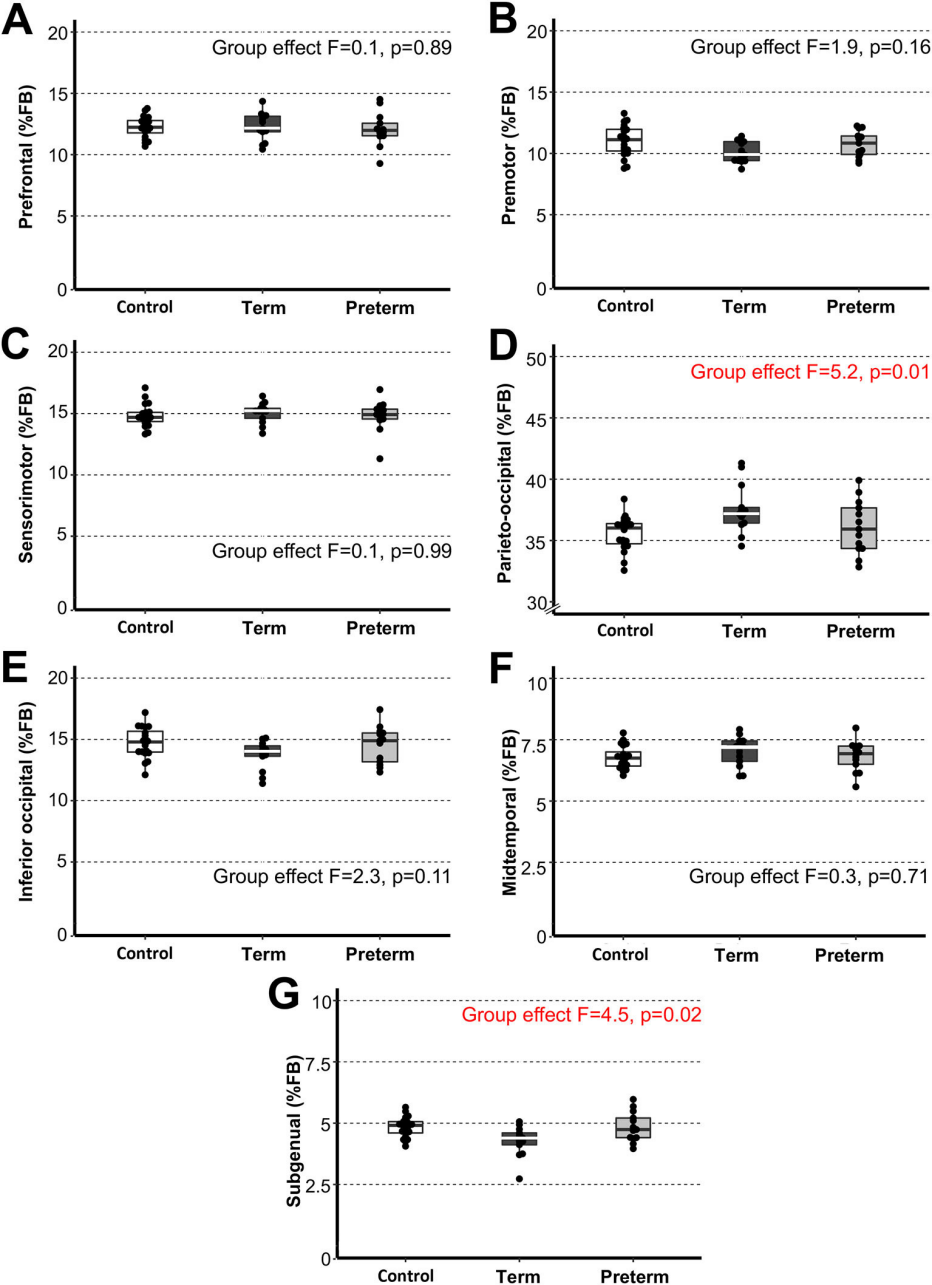
Sub-regional forebrain volumes. Graphs display individual *normalized* sub-regional volumes **(A–G)** as a % forebrain (FB) volume for the three study groups: (1) term-born controls (*n* = 20; white box), (2) term-born patients (*n* = 13; black box), and (3) preterm-born patients (*n* = 13; gray box). As noted in Methods section, forebrain volume was not available for 1/21 controls due to incomplete brain coverage, which accounts for final control group *n* = 20. We report no group differences for normalized sub-regional forebrain volumes with exception for normalized parieto-occipital **(D)** and subgenual **(G)** sub-regions between term-born patients and controls (*p* ≤ 0.01) and between patient groups (*p* ≤ 0.04), but not preterm patients and controls (*p* ≥ 0.42). See also Table 3 for complete statistics. The latter findings may be attributed to higher variability of data in premature patients and should be interpreted with caution. Dots represent individual data values, boxes represent the interquartile range (25–75%), and solid horizontal lines represent medians.

The ITK-SNAP volume estimation tool was used to obtain absolute volumes (cm^3^) of segmentation masks. Normalization was calculated as part of a whole to allow for better understanding as to how a particular sub-region of interest changes with respect to the whole (viz. total forebrain volume). Specifically, volumetric data of both CC and forebrain sub-regions were normalized as a % total forebrain volume (Figures 3, 4, respectively).

### Statistical Analysis

This study used original data from our previous pilot study (22). Since no prior information was available regarding brain findings in the selected cohort of infants with LGEA, we used a convenience sample size of 13 patients/group, based on the anticipated number of eligible infants at our institution and an estimated 50% enrollment rate. Statistical analyses were performed using the Statistical Package for the Social Sciences (SPSS, v.23.0; IBM Corporation, Armonk, NY), and normal distribution of all continuous variables was confirmed using the Shapiro-Wilk test. To account for the potential confounding variable of having subjects scanned at various ages throughout the first year of life, comparisons of sub-regional volumes between the three groups were assessed using a general linear model (GLM) univariate analysis with corrected age at scan as a covariate and Bonferroni adjusted *p*-values. Statistical significance was assessed at the α <0.05 level after conservative Bonferroni correction that accounted for multiple comparisons. In this report, we present differences between three groups studied. A comprehensive statistical report for absolute and normalized group differences is also condensed in a table format (Tables 2, 3).

**Table 2:** Analysis of sub-regional corpus callosum volumes.

	**(I)** **Genu & rostrum**	**(II)** **Rostral body**	**(III)** **Anterior midbody**	**(IV)** **Posterior midbody**	**(V)** **Isthmus**	**(VI)** **Splenium**
**CC SUB-REGIONAL ABSOLUTE VOLUMES (cm** ^**3**^ **)**						
Group differences	*F* = 15.06	*F* = 6.96	*F* = 19.11	*F* = 21.06	*F* = 22.89	*F* = 14.79
	*p* < 0.001	*p* = 0.002	*p* < 0.001	*p* < 0.001	*p* < 0.001	*p* < 0.001
**Individual group comparisons**						
C vs. T	*p* < 0.001	*p* = 0.01	*p* < 0.001	*p* < 0.001	*p* < 0.001	*p* < 0.001
C vs. P	*p* < 0.001	*p* = 0.002	*p* < 0.001	*p* < 0.001	*p* < 0.001	*p* < 0.001
T vs. P	*p* = 0.90	*p* = 0.60	*p* = 0.79	*p* = 0.49	*p* = 0.61	*p* = 0.41
**CC SUB-REGIONAL NORMALIZED VOLUMES (% TOTAL FOREBRAIN)**						
Group differences	*F* = 12.43	*F* = 1.94	*F* = 14.14	*F* = 15.04	*F* = 15.91	*F* = 11.20
	*p* < 0.001	*p* = 0.16	*p* < 0.001	*p* < 0.001	*p* < 0.001	*p* < 0.001
**Individual group comparisons**						
C vs. T	*p* < 0.001	*p* = 0.32	*p* < 0.001	*p* < 0.001	*p* < 0.001	*p* < 0.001
C vs. P	*p* < 0.001	*p* = 0.06	*p* < 0.001	*p* < 0.001	*p* < 0.001	*p* = 0.002
T vs. P	*p* = 0.94	*p* = 0.41	*p* = 0.70	*p* = 0.38	*p* = 0.69	*p* = 0.34

*Table summarizes statistical data from corpus callosum (CC) sub-regional volumetric analysis that included total of 6 sub-regions of CC (Figure 1): (I) genu & rostrum, (II) rostral body, (III) anterior mid-body, (IV) posterior mid-body, (V) isthmus, and (VI) splenium. Statistical results relate to graphs presented in Figure 3 that analyzed differences between three groups: healthy controls (C), and term-born (T) and preterm-born (P) patients following treatment for LGEA. Shaded boxes indicate statistically insignificant values. For details of statistical analysis, refer to Methods section*.

**Table 3:** Analysis of sub-regional forebrain volumes.

	**PFC**	**PM**	**SM**	**PO**	**IO**	**MT**	**SG**
**FOREBRAIN SUB-REGIONAL ABSOLUTE VOLUMES (cm** ^**3**^ **)**							
Group differences	*F* = 10.86	*F* = 12.24	*F* = 15.53	*F* = 5.31	*F* = 12.57	*F* = 10.34	*F* = 10.64
	*p* < 0.001	*p* < 0.001	*p* < 0.001	*p* = 0.01	*p* < 0.001	*p* < 0.001	*p* < 0.001
**Individual group comparisons**							
C vs. T	*p* < 0.001	*p* < 0.001	*p* < 0.001	*p* = 0.02	*p* < 0.001	*p* = 0.001	*p* < 0.001
C vs. P	*p* < 0.001	*p* = 0.001	*p* < 0.001	*p* = 0.01	*p* = 0.001	*P* < 0.001	*p* = 0.001
T vs. P	*p* = 0.98	*p* = 0.39	*p* = 0.94	*p* = 0.63	*p* = 0.25	*p* = 0.76	*P* = 0.10
**FOREBRAIN SUB-REGIONAL NORMALIZED VOLUMES (% TOTAL FOREBRAIN)**							
Group differences	*F* = 0.12	*F* = 1.93	*F* = 0.01	*F* = 5.16	*F* = 2.29	*F* = 0.34	*F* = 4.49
	*p* = 0.89	*p* = 0.16	*p* = 0.99	*p* = 0.01	*p* = 0.11	*p* = 0.71	*p* = 0.02
**Individual group comparisons**							
C vs. T	*p* = 0.70	*p* = 0.06	*p* = 0.90	*p* = 0.003	*p* = 0.05	*p* = 0.41	*p* = 0.01
C vs. P	*p* = 0.68	*p* = 0.39	*p* = 0.92	*p* = 0.42	*p* = 0.71	*p* = 0.77	*p* = 0.98
T vs. P	*p* = 0.99	*p* = 0.32	*p* = 0.98	*p* = 0.04	*p* = 0.13	*p* = 0.63	*p* = 0.02

*Table summarizes statistical data from forebrain (FB) sub-regional volumetric analyses that included total of seven sub-regions (Figure 2): (1) prefrontal cortex (PFC) that is comprised of orbitofrontal and dorsal prefrontal areas – the most anterior sub-regions of the forebrain, (2) premotor (PM), (3) sensorimotor (SM), (4) parieto-occipital (PO), (5) inferior occipital (IO), (6) midtemporal (MT), and (7) subgenual (SG) sub-regions. Statistical values relate to graphs presented in Figure 4 that analyzed differences between three groups: healthy controls (C), and term-born (T) and preterm-born (P) patients following treatment for LGEA. Shaded boxes indicate statistically insignificant values. For details of statistical analysis, refer to Methods section*.

## Results

In this report, we share quantitative analysis of T1-weighted data for six CC and seven forebrain sub-regional volumes among term- and preterm-born patients following LGEA repair (*n* = 13/group), and term-born controls (*n* = 21; Table 1).

### Sub-regional Analysis of Corpus Callosum

We previously reported disproportionately smaller total CC volume (as % of total brain volume) in both patient groups (11). Our novel analysis of total CC as a % of total forebrain volume matched these previous findings. Specifically, normalized total CC volumes showed significant group differences [F_(2,42)_ = 16.16, *p* < 0.001], with both term-born and preterm-born patients having smaller normalized total CC volumes compared to controls, with no differences between patient groups (graph not shown).

To evaluate sub-regional vulnerability of CC, we analyzed volumes of six CC sub-regions as illustrated in Figure 1A',B'. Statistical analysis of absolute values (cm^3^) between groups is summarized in Table 2. We normalized all six sub-regions of the CC as % total forebrain to see if any of the specific CC sub-regions in patients are smaller when compared to the forebrain as a whole. We report that normalized CC sub-regional volumes (as % forebrain volume; Figure 3) were significantly smaller in both patient groups compared to controls in all sub-regions except for the sub-region II (rostral body). For full statistical details, (see Table 2). As such, results implicate the rostral body, sub-region II of the CC to be least affected.

### Sub-regional Analysis of Forebrain

Our current T1-weighted analysis parallels the results of previously published T2-weighted data report (12) that showed proportionally smaller total forebrain (as % of total brain volume). Specifically, no group differences were observed in normalized volumes for total forebrain [F_(2,42)_ = 0.1, *p* = 0.90; graph not shown].

We extended the study to perform 3-D segmentation of seven forebrain sub-regions according to previous report in literature (24, 25), as illustrated in Figure 2C. Of the parcellated seven forebrain sub-regions, six are known to send homotopic projections via selective sub-regions of CC (26), as schematically color-coded in Figure 2C,D. In the absence of better methodology, presented results should be interpreted with caution due to linear parcellation methodological approach not fully aligned to functional connectivity (see section Study Limitations: Methodological Consideration). Statistical analysis of decreased absolute values (cm^3^) per sub-region in patients is summarized in Table 3. However, we only found group differences for normalized sub-regional volumes of the forebrain (as % total forebrain volume; Figure 4) for two forebrain sub-regions: parieto-occipital and subgenual sub-regions (term-born patients were different from both controls and premature patients). For full statistical details, (see Table 3). Future studies with more power and narrower clinical criteria for inclusion of premature patients should be considered.

## Discussion

Complex perioperative critical care with Foker process (14–16) in infants born with LGEA represents a standard of care. In this manuscript, we bring forth data with intention to bridge the gap in our understanding of such complex care's impact on brain development in infants born with LGEA. This work was built upon previous findings of disproportionally smaller total CC size in both term-born and premature infants following complex LGEA repair (11) and was extended to an investigation of homotopic-like sub-regional CC and forebrain volume analysis. Due to pilot study nature and methodological limitations of the forebrain segmentation in infancy, authors caution the readers against extrapolation of data until future studies confirm presented results.

### Homotopic-Like Analysis of Corpus Callosum and Forebrain Volumes

**Figure 5:**
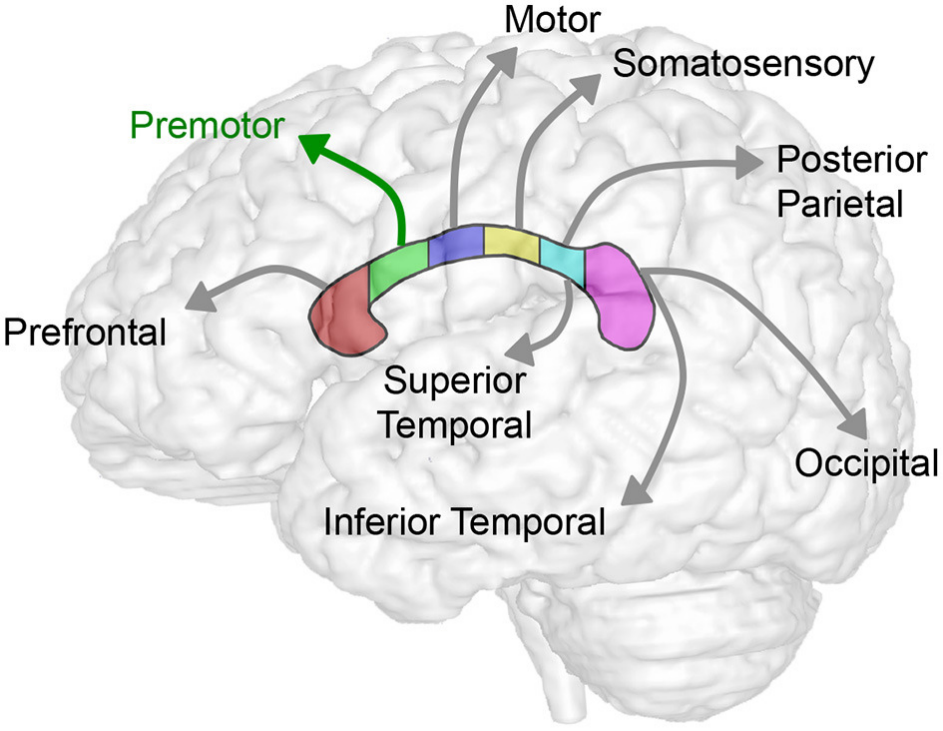
Summary schematics of corpus callosum to forebrain homotopic-like projections. Schematic illustrates homotopic projections between corpus callosum and the forebrain sub-regions. In this study, we showed consistent decrease in volumes across sub-regions of CC (Table 2; Figure 3) and forebrain (Table 3; Figure 4) in selected group of infants following complex perioperative critical care for long-gap esophageal repair. Global decrease of the volumes appears least affected in the region II of the CC (viz. rostral body of the CC) known to project to the premotor cortex (green arrow) following esophageal repair with Foker process. Interestingly, a disproportionally smaller normalized volume of subgenual sub-region of the forebrain (not illustrated; see Figure 2C) does not have any direct homotopic connections via CC.

Our current data of sub-regional CC size implicates globally smaller CC in premature and term-born patients. The uniformly decreased normalized CC volume with respect to the whole forebrain [with the exception of the sub-region II of the CC (viz. rostral body)], may implicate delayed and/or impaired maturation of white matter tracts in the setting of LGEA as a congenital anomaly and/or result of LGEA complex perioperative treatment. As such, CC volume analysis may serve as an early marker of brain (mal)adaptations of brain size in the selected group of infants born with LGEA undergoing complex perioperative critical care with Foker process (14–16). Our volumetric analysis showed disproportionally larger normalized volume in the rostral body (sub-region II of the CC) known to connect to the pre-motor cortex, indicating that this region may be least likely affected (schematics summary in Figure 5) in the context of globally smaller brain in selected cohort of infants (22). The anterior callosal fibers such as those projecting from the rostral body have been shown to interconnect to the frontal lobe and relate motor information (6). Larger normalized rostral body findings, and homotopic-like linkages between the rostral body and the premotor area, which was also shown to have potentially altered growth trajectories for the patient groups, may indicate possible protection in this area of the forebrain, an interesting and unexpected finding given the previously established delay in motor development in critically ill infants (46). Conversely, the more adversely affected posterior CC fibers connecting the parietal, temporal, and occipital lobes, the greater likelihood of (mal)adaptations in integration of sensory information (6) might be encountered. To our knowledge, no studies yet have evaluated the neurodevelopmental outcomes in either term- or preterm-born infants following complex perioperative critical care with Foker process for LGEA repair. As such, future clinical evaluations that include motor and sensory assessments should be used to correlate neuroanatomical findings to the neurodevelopmental implications.

Although no previous studies have investigated sub-regional CC development in the setting of complex perioperative critical care, previous work has shown that the more posterior sub-regions of the CC (viz. anterior midbody, posterior midbody, isthmus, and splenium; Figure 1) are likely to have smaller surface areas, decreased volume of interhemispheric fiber tracts, and reduced fractional anisotropies in very premature babies (41). This is consistent with our findings of decreased volumes in such posterior areas, and possible protective effects in the more anterior rostral body sub-region (sub-region II of CC). Importantly, as CC myelination occurs anteriorly to posteriorly (10), such findings may indicate the posterior sub-regions of the CC are more vulnerable to adverse early life events in the context of the complex perioperative critical care for LGEA repair.

Our current sub-regional analysis of normalized forebrain volumes failed to identify vulnerable sub-region of the forebrain in homotopic-like relation to CC sub-regions. As such, results indicate that uniformly decreased sub-regional forebrain volumes contribute to the global decrease in brain size (22) in the selected pilot cohort of infants following LGEA repair. Interestingly, a disproportionally smaller normalized volume is reported only for the subgenual sub-region of the forebrain (Figure 2C), which does not have any homotopic connections with the CC (Figure 5), and suggest that, possibly - limbic circuits may be adversely impacted following complex perioperative critical care for LGEA repair.

### Underlying Brain Mechanisms

The disproportionately smaller CC sub-regional volumes may indicate hypo or incomplete myelination in term-born and premature patients. Whether this is due to decreased oligodendrocyte cells, decreased axonal density, or degradation of the myelin, remains unclear. It should be noted, that previous studies have linked prematurity to focal non-cystic white matter injuries in nearly half of premature newborns with MRI data, and much of this white matter vulnerability has been linked to late oligodendrocyte progenitor cells/ subplate cells (47). Whether this is true of the underlying pathology of term-born infants undergoing complex perioperative care warrants future studies. Previous literature has also reported term-born infants undergoing complex care, including those both with and without major cardiac anomalies, are more likely to have white matter lesions (cardiac and non-cardiac cases) and lower fractional anisotropy in the CC (cardiac cases) (48, 49). Whether the decreased CC size in our sample population of infants with LGEA reflects preexisting brain abnormalities, risk of abnormal development in the setting of perioperative critical care, or a double-hit etiology of brain injury both pre and peri-Foker process, as previously discussed (18), remains unknown. Our most recent preliminary data published as a small case report series (18) are in support of possible double-hit etiology of brain injury (pre-Foker and peri-Foker process) in both premature and term-born infants with LGEA. Future studies in this vulnerable infant population should also include diffusion tensor imaging (DTI) and tractography to shed light on the possible mechanisms affecting white matter microstructural integrity during infancy, a critical age for the white matter maturation and global brain growth.

### Neurobehavioral Significance

The exact neurological effects and long-term neurobehavioral impact of complex perioperative care for LGEA repair remain unknown. Previous reports indicate that infants with non-cardiac congenital anomalies that undergo complex perioperative care are at an increased risk for abnormal brain development and poor long-term outcomes (9, 48). More broadly, as the largest white matter tract in the brain that serves to connect and facilitate cross-talk between the two hemispheres, abnormal development of the CC in infancy may lead to altered cognitive/executive function that persists into childhood and adolescence (50). Prematurity, white matter impairment, and decreased CC volume have also all been associated with poor executive functioning and language outcomes (51–53). Since functional implications of reported structural differences remains unknown in infants following complex perioperative critical care for LGEA repair, investigation into clinical correlations and long-term neurodevelopmental outcomes represents the next steps in clinical research investigation.

### Study Limitations

#### Methodological Considerations

As the infant forebrain is undergoing constant rapid myelination, a clear parcellation scheme based on functional significance has yet to be developed. Rather, a previously validated parcellation scheme based on distinct anatomical features rather than functional significance was utilized. Homotopic-like connections between the forebrain and CC were then established according to existing literature (26). In other words, since the parcellation was not performed based on the true connectivity between the corpus callosum and the forebrain, current parcellation scheme does not fully reflect functional relation. Future studies following technical automated segmentation advancement of the forebrain to more defined structures, could be used to assess a more structurally/functionally precise method of understanding homotopic CC-forebrain relationship.

#### Study Timing

Previous literature has estimated robust linear postnatal growth of cortical gray-matter volume in the first year of life, and comparatively linear—but slower growth of cortical white-matter volume (54). Future studies should include cross-sectional analysis to account for linear growth rates differences between gray and white matter in the first year of life.

#### Study Size

Small sample size and a lack of subjects scanned at older time points (>8 months of age) pose potential limitations. Age-related changes were taken into consideration by using a general linear model with gestation-corrected age as a covariate, but future studies with larger sample size and a more uniform age distribution are necessary.

#### Control Group

We only used term-born control infants that served as a comparison to normative size. However, future studies should consider additional controls such as (1) a cohort of non-cardiac LGEA that underwent alternative treatment to the Foker process, (2) a cohort that received only prolonged sedation without surgery, and (3) a cohort of premature infants that received no medical care.

#### Sex Differences

Previous studies remain inconsistent, with some reporting no sex differences in CC area, and other reporting larger normalized female CC volumes (6, 55). Although we had equal distribution of sex/patient group, our term-born control group consisted predominately of male infants. Future studies should have enough power to allow for sex differences analysis of both CC volumes and its homotopic-like projections to the forebrain.

#### Pre-existing Findings

Care was taken to exclude infants with previously existing neurological disease or brain abnormalities. However, infants were not given MRI scans prior to treatment, so it is impossible to know if size (mal)adaptations in CC/forebrain size pre-existed to the complex perioperative critical care, as is the case with pre-surgical differences in CC volume of patients with congenital heart defects that has been shown to worsen post-surgery (56).

## Conclusions

Our novel sub-regional quantitative analysis strengthens previous observations of qualitative brain atrophy and quantitatively decreased global and regional brain size in a unique pilot cohort of term-born and preterm-born infant patients *without* previously recognized neurological injury or insult. Future longitudinal studies should include diffusion tractography of the CC and forebrain in further evaluation of what appears to represent *posteriorly* diminished homotopic-like CC/forebrain volume, and protection of rostral body/premotor regions, following complex treatment of infants born with LGEA.

## Data Availability Statement

The raw data supporting the conclusions of this article will be made available by the authors, without undue reservation.

## Ethics Statement

The studies involving human participants were reviewed and approved by Boston Children's Hospital Institutional Review Board (IRB-P000007855). Written informed consent to participate in this study was provided by the participants' legal guardian/next of kin.

## Author Contributions

CM and DB: the conception and manuscript design. MK, CM, and DB: acquisition. MK, DZ, and DB: analysis. MK and DB: drafting the article. All authors: interpretation of data, critical revision for important intellectual content, final approval of the version to be published, and accountable for all aspects of the work in ensuring that questions related to the accuracy or integrity of any part of the work are appropriately investigated and resolved.

## Funding

This work was supported by the NIDA K08 DA035972-01 (to DB) and 2017 *Trailblazer Award* from the Department of Anesthesiology, Critical Care and Pain Medicine, Boston Children's Hospital (to DB).

## Author Disclaimer

The content of this article is solely the responsibility of the authors and does not necessarily represent the official views of the National Institutes of Health.

## Conflict of Interest

The authors declare that the research was conducted in the absence of any commercial or financial relationships that could be construed as a potential conflict of interest.

## Publisher's Note

All claims expressed in this article are solely those of the authors and do not necessarily represent those of their affiliated organizations, or those of the publisher, the editors and the reviewers. Any product that may be evaluated in this article, or claim that may be made by its manufacturer, is not guaranteed or endorsed by the publisher.

## Abbreviations

AC-PC: anterior commissure—posterior commissure
A-P: anterior—posterior
CA: corrected age
CC: corpus callosum
CSF: cerebrospinal fluid
FAST: FMRIB's Automated Segmentation Tool
FSL: FMRIB Software Library
FB: forebrain
DF: dorsal prefrontal sub-region
IO: inferior occipital sub-region
MT: midtemporal sub-region
OF: orbitofrontal sub-region
PFC: prefrontal cortex combined sub-region
PM: premotor sub-region
PO: parieto-occipital sub-region
SG: subgenual sub-region
SM: sensorimotor sub-region
GA: gestational age
GLM: general linear model
IUGR: intrauterine growth restriction
LGEA: long-gap esophageal atresia
MRI: magnetic resonance imaging
SGA: small for gestational age
SPSS: Statistical Package for the Social Sciences
TEF: Tracheo-esophageal fistula.
